# Study on Microwave Curing of Unsaturated Polyester Resin and Its Composites Containing Calcium Carbonate

**DOI:** 10.3390/polym14132598

**Published:** 2022-06-27

**Authors:** Qiufeng Mo, Yifeng Huang, Lanyu Ma, Wenqin Lai, Yihua Zheng, Yanming Li, Mengxue Xu, Zhimin Huang

**Affiliations:** Guangxi Academy of Sciences, Nanning 530000, China; 18078131362@163.com (Q.M.); hyifeng123@163.com (Y.H.); mlanyu@163.com (L.M.); laiwq9623@163.com (W.L.); yhzhengjlu@126.com (Y.Z.); lym810555@163.com (Y.L.)

**Keywords:** unsaturated polyester resin, microwave, curing, calcium carbonate, microwave power

## Abstract

Microwave curing technology has been widely used in resin and its composite materials. In order to study its effect for curing unsaturated polyester resin (UPR) composites containing calcium carbonate (CaCO_3_) filler, this paper first investigated the influence of microwave power and microwave irradiation time on the curing characteristics of UPR. Then, CaCO_3_ particles were added to the UPR to investigate the microwave curing effect of the UPR composites containing the CaCO_3_. The results showed that microwave irradiation could heat the UPR sample evenly, and rapidly cause the chain growth reaction, thus greatly shortening the curing time. The curing degree and products of the samples after microwave curing were consistent with that of the thermal curing. The addition of CaCO_3_ particles could increase the heating rate of the UPR composites, which would accelerate the curing rate of the UPR. However, higher microwave power could lead to pore defects inside the UPR composites with higher CaCO_3_ content, resulting in a lower strength. Thus, the compactness of the samples should be improved by reducing the microwave power and prolonging the microwave treatment time.

## 1. Introduction

Unsaturated polyester resin (UPR) is a linear polymer compound with multifunctional groups. There is a large number of ester groups (-COOR) and carbon–carbon double bonds (C=C) on the unsaturated polyester molecular chain, and the two ends of the chain are carboxyl groups (-COOH) and hydroxyl groups (-OH), respectively [1,2,3,4]. Due to its good mechanical properties and technical performance after curing, UPR is widely used in artificial stone, coating, casting, glass fiber reinforced plastics, and other industries [5,6].

The curing of UPR usually requires to be initiated by an initiator, such as methyl ethyl ketone peroxide and cyclohexanone peroxide. Then, a free-radical copolymerization reaction between the C=C in the unsaturated polyester molecular chain and vinyl monomers occurs, forming a polymer with a stable three-dimensional network structure [7,8,9]. However, the initiators must be decomposed and activated under the conditions of heating or accelerator induction. Otherwise, its decomposition speed cannot meet the curing process requirements of the UPR [10,11,12]. At present, the main curing method of the UPR is a traditional thermal curing process. However, the UPR is a poor thermal conductor and unable to be cured evenly inside and outside using the thermal curing method. In addition, the curing rate of this method is slow, and its energy consumption is large [13]. Different from the thermal curing method, microwaves are electromagnetic energy wave that has a strong penetrating ability. Therefore, microwaves can radiate the whole reactive material and change the thermodynamic function of the entire system, so as to achieve the effect of heating the material inside and outside at the same time. In other words, the material is uniformly heated under microwave irradiation without the heating hysteresis effect [14,15]. In addition, microwave irradiation can make the material molecules move, collide and rub at a high speed in a short period of time, thereby heating up heating up the materials rapidly through dielectric loss, thus reducing the activation energy of the reaction and the molecular bond strength, greatly accelerating the reaction speed and shortening the reaction period [16,17,18]. It is known that the UPR contains a large number of polar groups, such as -OH and -COOH, which endow the UPR with a wave-absorbing ability. Therefore, the microwave curing technology of the UPR has received much attention.

A. R. Rahmat et al. [19,20] compared microwave curing and thermal curing of UPR and the unsaturated polyester/aramid composites. It was found that microwave curing was much faster than thermal curing, and the microwave cured composite obtained a higher crosslinking density and better mechanical properties. Feng et al. [21] conducted numerical simulation and experimental research on microwave curing of UPR artificial marble blocks. The results showed that microwave hardened artificial marble blocks achieved a better overall performance than naturally hardened ones in terms of hardness and strength, which enhanced industrial production of the UPR artificial marble efficiently. Adefemi et al. [22] studied the tensile strength of aluminum and carbon black reinforced unsaturated polyester composites cured by microwave and conventional thermal methods, respectively. The results indicated that the tensile strength of the composites cured via microwave was improved as compared to the thermal method.

In the various application fields of the UPR, the quality defects of the products are largely related to their curing process [23,24]. Hence, it will be of great use to conduct in-depth discussion and research on the influencing factors and curing characteristics of the microwave curing for the UPR. In addition, calcium carbonate is generally used as an important filling material in UPR-based composite materials. For example, the proportion of CaCO_3_ in coatings reaches 30–50%, while the proportion of CaCO_3_ in artificial stone reaches as high as 80–90% [25,26,27,28]. Therefore, this paper firstly studied the microwave curing characteristics of the UPR, and then introduced the CaCO_3_ particles to investigate the effect of CaCO_3_ fillers on the microwave curing process of the UPR composites, which would provide basic data and theoretical guidance for the microwave curing of the UPR composites.

## 2. Experimental

### 2.1. Materials

**Figure 1:**
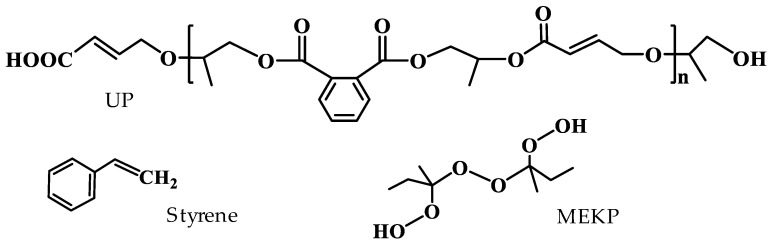
Molecular structures of UPR, styrene, and MEKP.

Figure 1 shows the molecular structure of the unsaturated polyester (UP), styrene, and the initiator of methyl ethyl ketone peroxide (MEKP, C_8_H_18_O_6_). The UPR was purchased from Shanghai Jiyi Chemical Co., Ltd., in which UP and styrene were about 65% and 35% by weight, respectively. The MEKP was supplied by Shanghai McLean Biochemical Technology Co., Ltd., Shanghai, China. Calcium carbonate powder was less than 800 mesh. During the curing process, a silicone material was used as the container mold of UPR and UPR composites. The size of the samples is 50 × 50 × 45 mm^3^. The microwave-generating device was a household microwave oven (Midea, M1-230E, Foshan, China). The microwave frequency of the microwave oven was 2450 MHz, and its five-level output powers were 136 W, 264 W, 440 W, 616 W, and 800 W, respectively.

### 2.2. Curing of UPR Composites

#### 2.2.1. Sample Preparation

CaCO_3_ powder was added to the UPR under stirring condition at 1500 r/min for 10 min. Next, 1.0 wt.% of MEKP was added, continuously stirring for 10 min at 1000 r/min and standing for 60 min to remove air bubbles. The uncured UPR composites with CaCO_3_ mass percentages of 0%, 10%, 30%, and 50%, respectively, were obtained. The UPR composite without CaCO_3_ particles was named the UPR sample. The initial temperature of the samples was 20 ± 2 °C.

#### 2.2.2. Thermal Curing

The UPR composites prepared in step (1) were transferred to the silicone mold and then cured in an oven at 90 °C for 120 min. At the same time, a thermometer was used to monitor the temperature data of the sample center. After the temperature was lowered to room temperature (20 ± 2 °C), the samples were ready for test.

#### 2.2.3. Microwave Curing

The uncured samples were placed in the microwave oven, and the monofactor variable method was adopted to investigate the influence of microwave power and microwave irradiation time on the uncured UPR composite samples. After microwave treatment, the temperature data of the samples was continuously monitored with the thermometer.

**Figure 2:**
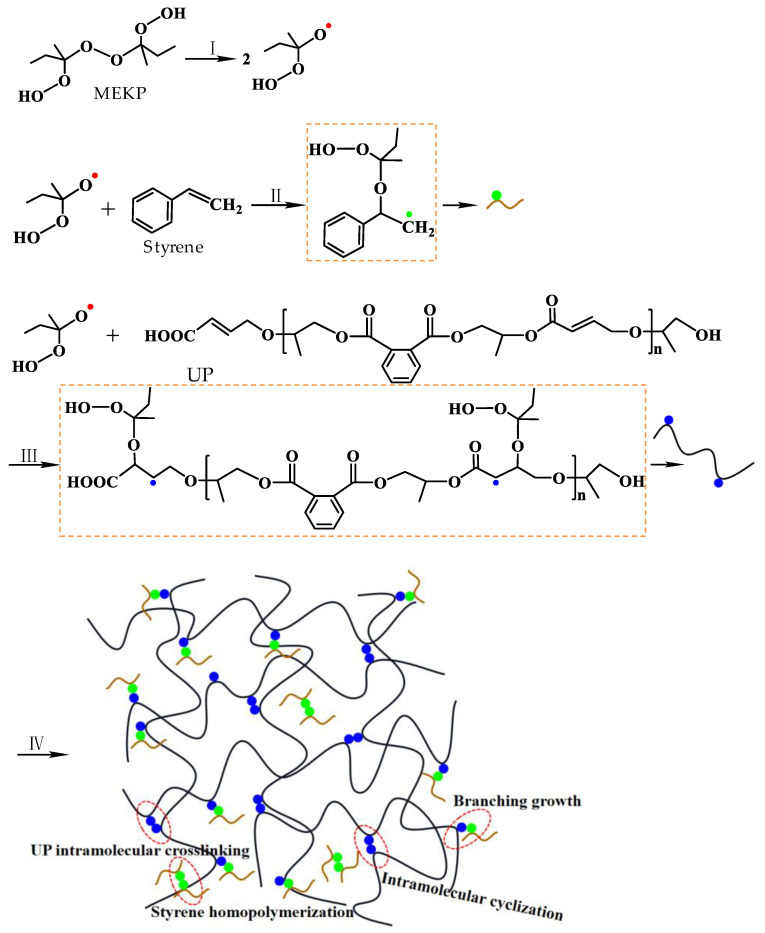
Schematic diagram of UPR curing.

The curing process of UPR was shown in Figure 2. The unstable MEKP was first decomposed under heating or microwave and released reactive oxygen radicals (reaction I), which could rapidly act on the C=C of the UP molecular chains and styrene molecules. Then, the C=C was opened to generate new free-radicals with unpaired isolated electrons (reaction II and reaction III). Subsequently, the free-radicals of the UP and styrene monomers quickly participated in the cross-linking polymerization reaction and forming a three-dimensional structure. Shown in reaction IV, these reactions could be the branching growth between styrene and UP monomers, the homopolymerization of styrene monomers, and the intramolecular crosslinking and intramolecular cyclization of the UP monomers [23,29,30].

### 2.3. Characterizations

The surface temperature of the samples was measured by using a pocket thermal imager (FLUKE PTi120). The cross-sectional view was observed by a microscope device (ZQ-601). In order to facilitate the observation of the pore distribution inside the samples, each of the samples was first colored with a blue marker before the observation. The compressive strength of the samples was conducted by an electronic universal testing machine (WDW-300, China). The test speed was 5 mm/min, and all the compressive strength of the UPR composites samples were repeated six times. The differential scanning calorimetry (DSC, Q20, TA Instruments Company, USA) measurements were carried out to investigate the curing behaviors of the UPR sample, and its curing degree after the thermal curing and microwave curing was compared. The test temperature ranged from 40 °C to 300 °C with a heating rate of 10 °C·min^−1^ under a nitrogen atmosphere. The nitrogen flow was 30 mL/min. The degree of cure (α) was calculated by Equation (1) [20].

(1) $$\alpha =\frac{H_{T}-H_{R}}{H_{T}}\times 100\%$$

where H_T_ was the total heat of reaction (J/g), H_R_ was the heat of residual reaction (J/g).

The solidification of the UPR was evaluated by Fourier-transform infrared spectroscopy (FTIR, Nicolct IS 10, ThermoFisher, Waltham, MA, USA) with a wavenumber range of 550–4000 cm^−1^. The scanning step size and scanning times were 2 cm^−1^ and 32 times, respectively. The absorption peak near 1716 cm^−1^ (caused by the stretching vibration of carbonyl group) was used as a reference standard to calculate the conversion rate of the UPR under the two curing methods. The conversion rate of the UPR was calculated by Equation (2).

(2) $$\alpha_{P}=1-\frac{A_{981}^{s}/A_{1716}^{s}}{A_{981}^{l}/A_{1716}^{l}}$$

where α_P_ was the conversion rate of the UPR, $A_{981}^{s}$ was the area of the absorption peak of the cured UPR at 981 cm^−1^, $A_{1716}^{s}$ was the area of the absorption peak of the cured UPR at 1716 cm^−1^, $A_{981}^{l}$ was the area of the absorption peak of the liquid UPR at 981 cm^−1^, and $A_{1716}^{l}$ was the area of the absorption peak of the liquid UPR at 1716 cm^−1^.

## 3. Results and Discussion

### 3.1. Microwave Curing Characteristics of UPR

**Figure 3:**
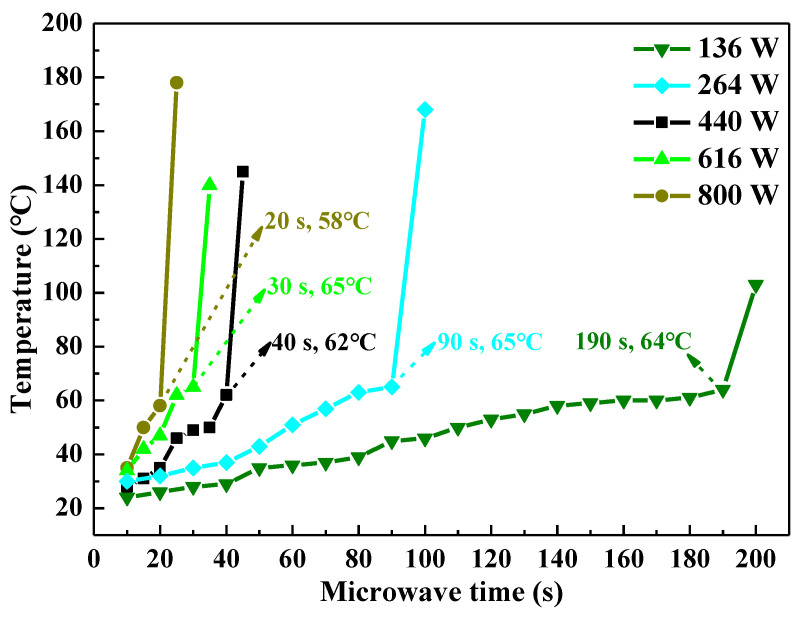
Influence of microwave power and microwave irradiation time on temperature of UPR.

Figure 3 displays the influence of microwave power and microwave irradiation time on the temperature of the UPR samples. Raising the microwave power and increasing microwave irradiation time could increase the temperature of the UPR samples. When the microwave power changed from 136 W to 800 W, the temperature of the samples suddenly and sharply increased after being irradiated by microwave for about 200 s, 100 s, 45 s, 35 s, and 25 s, respectively. It indicated that the UPR produced the free-radical copolymerization reaction during this period, and the system rapidly released a large amount of heat. In addition, it could be inferred that the self-accelerating decomposition reaction of the initiator MEKP only occurred when the temperature of the samples exceeded 65 °C [31,32].

**Figure 4:**
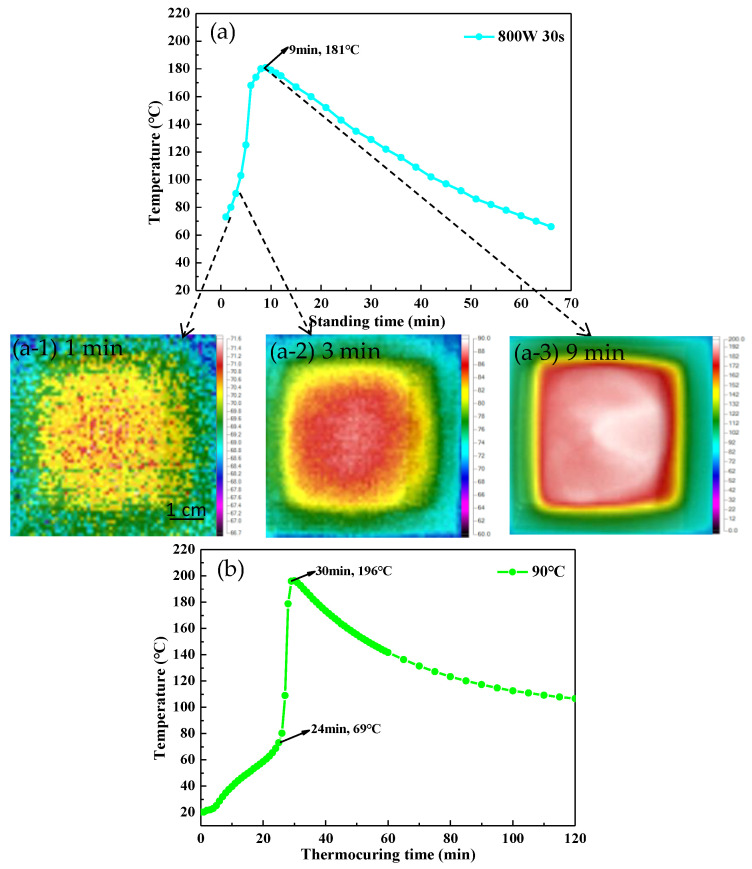
(**a**) Temperature of the UPR sample with increasing standing time after microwave irradiation under 800 W for 30 s. (**b**) Temperature of the UPR sample with a thermocuring time at 90 °C for 120 min.

In order to further analyze the curing process of UPR under microwave treatment, the temperature of the samples after microwave irradiation at 800 W for 30 s was monitored, and the temperature data were shown in Figure 4a. After microwave irradiation for 30 s, with the extension of standing time, the temperature of the sample increased rapidly and reached a maximum of 181 °C at 9 min. At that time, the addition polymerization reaction of the system was accelerated, a three-dimensional network was formed, and a gel phenomenon occurred [33]. Subsequently, the temperature showed an approximately linear downward trend, indicating that the sample can enter the stage of chain growth reactions after 30 s of microwave irradiation. In addition, it can be seen from the thermal imaging photos that the surface temperature of the sample was uniform at different reaction stages (Figure 4a-1–a-3). It demonstrated that the microwave radiation could make the UPR sample evenly heated, so that the system was cured as a whole. In contrast, it can be seen from Figure 4b that the temperature curve of the sample under thermostatic heat treatment showed three obvious stages. In the first stage, the temperature of the sample increased slowly due to the heat transfer. Eventually, it entered the stage of chain growth reactions after being heated for about 24 min, which was much slower than that of the microwave curing method. In this stage, the polymerization rate increased, and the temperature rises rapidly. After 30 min the system entered the stage of chain termination, and its temperature began to drop slowly. It revealed that microwave curing can greatly shorten the curing time of UPR.

**Figure 5:**
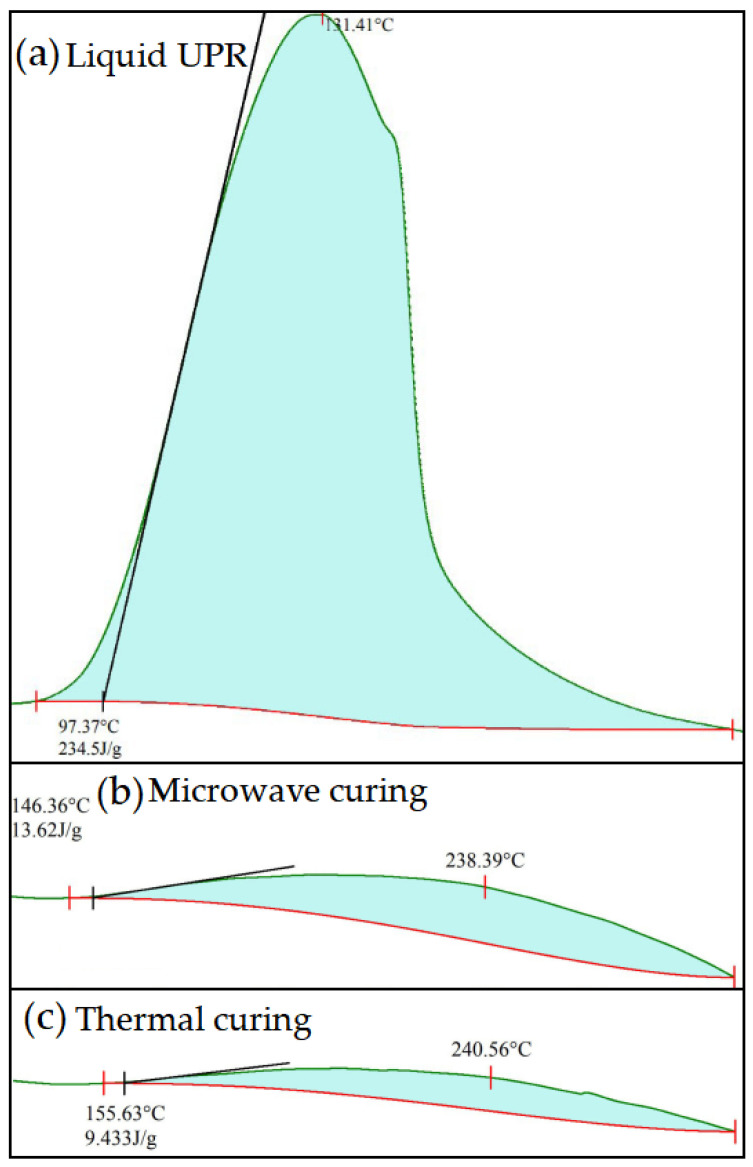
DSC curves of liquid UPR and cured samples with microwave curing and thermal curing, respectively.

Figure 5 shows the DSC results of the liquid UPR and cured samples under the microwave curing (800 W, 30 s) and thermal curing (90 °C, 120 min), respectively. It can be seen from Figure 5 that the DSC curve of the liquid UPR showed a large exothermic peak after being heated to 97 °C, and the curing absorption enthalpy of the liquid UPR was 234.5 J/g. After microwave curing, the enthalpy value of the sample decreased to 13.62 J/g. The absorption enthalpy value of the sample cured through thermal curing was 9.43 J/g. As shown in Table 1, according to Equation (1), the α of two cured samples were very close: 94.19% and 95.98%, respectively. It indicated that the UPR curing effect of microwave curing with 800 W for 30 s was consistent with that of thermal curing at 90 °C for 120 min.

**Figure 6:**
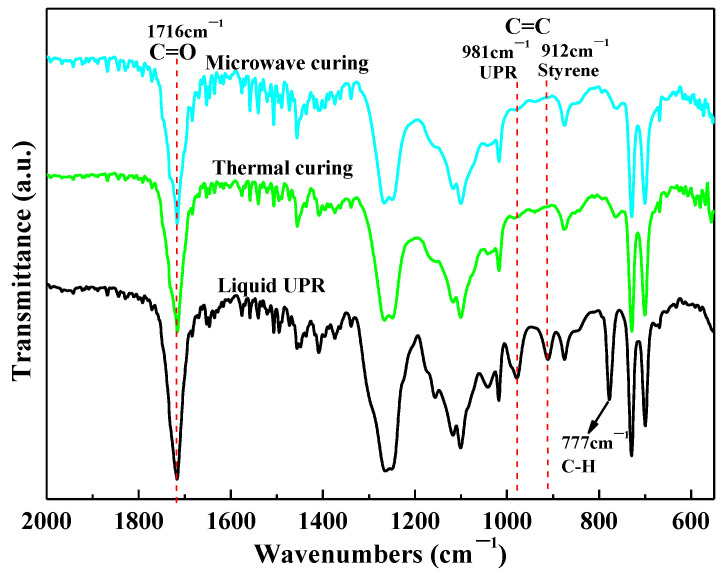
FTIR spectra of liquid UPR and cured UPR samples with thermal curing and microwave curing.

Figure 6 presents the FTIR spectra of the liquid UPR and its cured samples under thermal curing and microwave curing (800 W, 30 s), respectively. There were two obvious characteristic peaks at 981 cm^−1^ and 912 cm^−1^ detected on the liquid UPR sample, which were attributed to stretching vibration of C=C in the UP and styrene, respectively [20]. After being cured, the spectra of the two samples cured by thermal curing and microwave curing showed no obvious difference. It indicated that the structures of the cured products obtained by these two curing methods were approximately the same. Specifically, the two absorption peaks of C=C were greatly weakened. In addition, the absorption peak at 777 cm^−1^ related to the out-of-plane bending vibration of C-H on the benzene ring was also weakened. Combined with the curing mechanism of the UPR (Figure 2), it can be inferred that almost all the C=C bonds in UP reacted and a part of styrene volatilized during the curing process of the UPR. The conversion rates of the UPR (α_P_) calculated by Equation (2) were shown in Table 2. The results showed that the conversion rate of the C=C in UP under microwave curing was close to that of thermal curing. These results explain the equivalent curing effects of the two methods, which is consistent with the DSC results.

### 3.2. Microwave Curing Characteristics of UPR Composites with CaCO_3_

**Figure 7:**
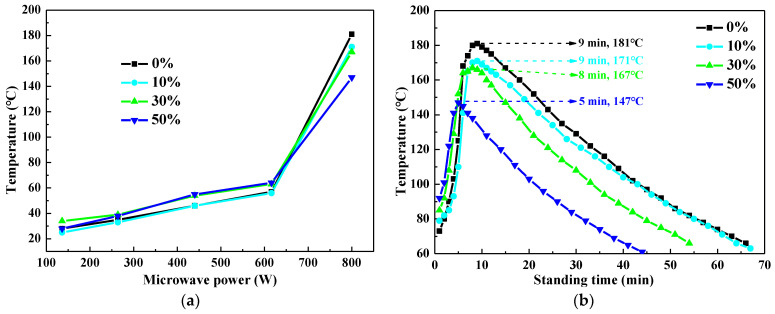
(**a**) Temperature of the UPR composites containing CaCO_3_ under different microwave powers for 30 s. (**b**) Temperature curves of the UPR composites after microwave irradiation under 800 W for 30 s.

Figure 7 shows the heating curves of UPR composites containing different CaCO_3_ contents after microwave irradiation for 30 s. When the microwave power was less than 616 W, microwave irradiation for 30 s was not enough to initiate the self-accelerating decomposition reaction of the MEKP (Figure 7a). In addition, the temperature of UPR composites generally increased with the CaCO_3_ content under the same microwave power. The reason was that the microwave heating rate was related to the microwave power absorbed by the material per unit volume, the density and the specific heat capacity of the material [34,35]. The density and specific heat capacity of CaCO_3_ are higher than the UPR, so it can be inferred that CaCO_3_ has a stronger wave-absorbing ability than the UPR. There, samples with higher content of CaCO_3_ showed higher temperatures under the same microwave irradiation. Figure 7b displays the temperatures of the UPR composites at different standing times after 30 s of microwave irradiation (800 W). With the increasing of the CaCO_3_ content, the samples reached the highest temperature after standing for 9 min, 9 min, 8 min and 5 min, respectively. It can be concluded that adding CaCO_3_ particles into the UPR can shorten the time required to enter the stages of chain growth reactions and chain termination, reducing the curing time of the UPR. However, the lower corresponding UPR content means the less the number of the free-radical monomers available for reaction in the UPR composites. Therefore, as the CaCO_3_ content increased, the total amount of reactions decreased, leading to the decrease of total exotherm and the maximum temperature of the UPR composites.

**Figure 8:**
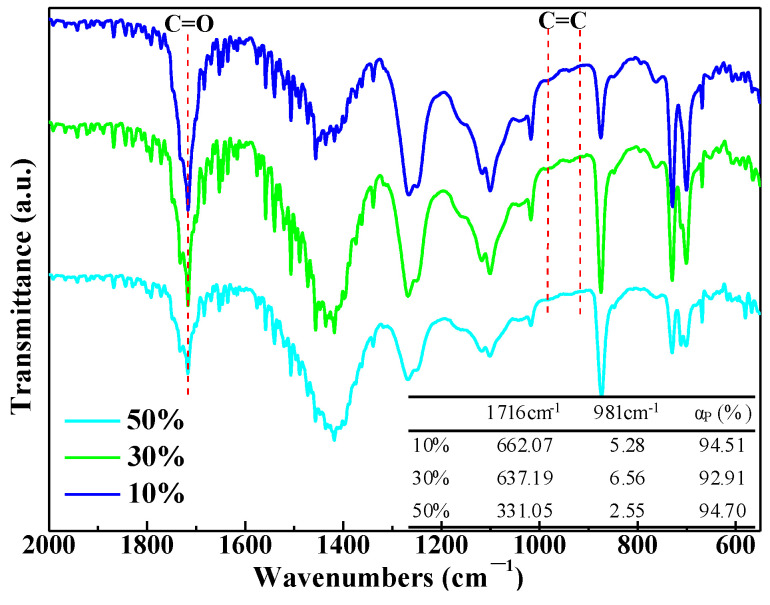
FTIR spectra and C=C conversion rate of the UPR composites under 800 W microwave for 30 s.

The cured UPR composites containing CaCO_3_ particles are analyzed by FTIR, and the results are shown in Figure 8. Similar to the UPR sample, two absorption peaks corresponding to the C=C in the spectra of three UPR composites were greatly weakened. Based on Equation (2), the conversion rates of the C=C in UP reached 92%. It indicated that the curing degree of the UPR composites containing CaCO_3_ is close to that of the UPR sample. The presence of the CaCO_3_ particles had no substantial effect on the cured products of the UPR using microwave irradiation.

**Figure 9:**
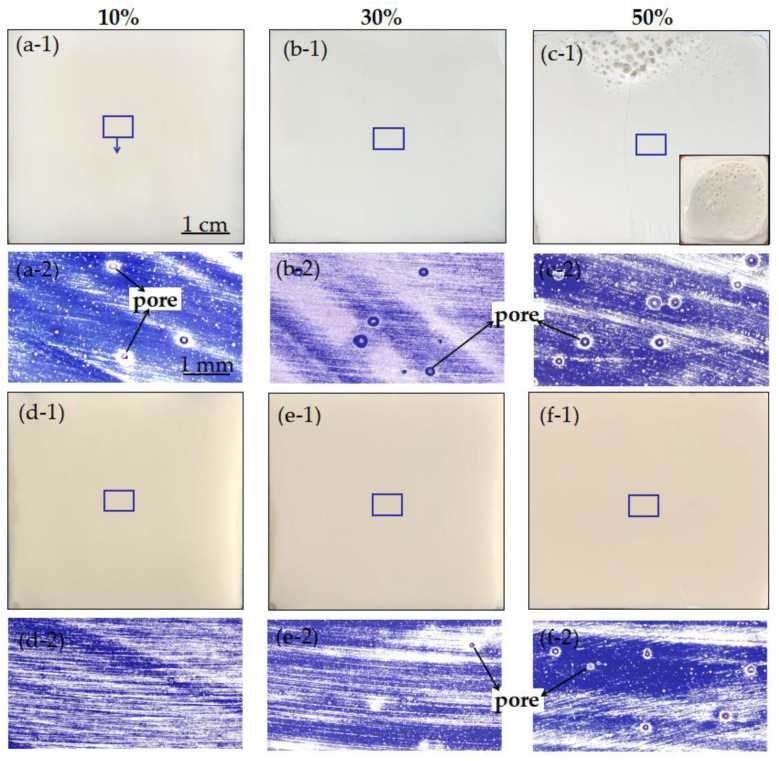
Cross-sectional view of the cured UPR composites under microwave curing (**a-1–c-2**) and thermal curing (**d-1–f-2**).

Figure 9 displays the cross-sectional view of the cured UPR composites under the thermal curing (90 °C, 120 min) and microwave curing (800 W, 30 s). As shown in Figure 9a-1,b-1, under the macroscopic cross-sectional view, there were no visible pores on the samples with 10–30% CaCO_3_. However, via the microscopic morphology, some pores (100–200 μm) were found on the sample with 10% CaCO_3_ (Figure 9a-2). A few pores with a size ranging from 100 to 300 μm were distributed on the sample with 30% CaCO_3_ (Figure 9b-2. As shown in Figure 9c-1, there were a large number of visible pores near the surface of the sample with 50% CaCO_3_. It was also found that the volume of the sample expanded significantly during the curing process, and lots of pores were visible on its surface (Figure 9c-1 (inset)). The size of these pores generally decreased from top to bottom (Figure 9c-2). Based on these results, it can be seen that the temperature of the sample with 50% CaCO_3_ increased the fastest (Figure 7), yet the density of the sample was also the largest, which could trap a large amount of gas (styrene, air) in the system. A large number of defects were eventually formed and remained inside the sample, leading to a decrease in the strength properties of the sample.

As shown in Figure 9d-1–f-1, no obvious pores were seen in the macroscopic cross-sectional view of the thermally cured samples. However, under the same thermal curing conditions, with the increasing of the CaCO_3_ content, more and more micropores remained inside the cured samples (Figure 9d-2–f-2). The size of the pores also increased, and the largest size of the pores was around 150 μm in the sample with 50% CaCO_3_. It was found that the internal defects of the samples cured at 90 °C for 120 min were less than that of the samples treated by 800 W microwave for 30 s. These results demonstrated the content of CaCO_3_ in the UPR composites and the curing method has a great influence on their compactness.

**Figure 10:**
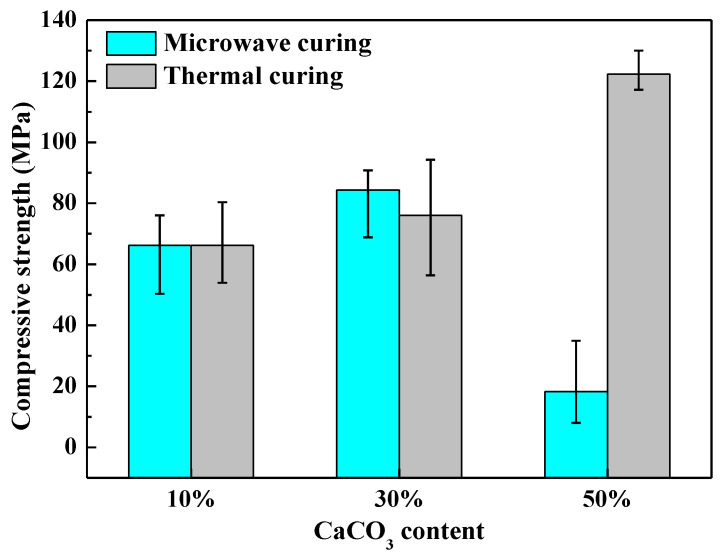
Compressive strength of the cured UPR composites under two curing methods.

Figure 10 shows the compressive strength of the cured UPR composites under two curing methods. After microwave curing (800 W, 30 s), the compressive strength of the samples (10%, 30%, and 50% CaCO_3_) was about 66 MPa, 84 MPa, and 18 MPa, respectively. Compared with the compressive strength of the cured samples using the traditional thermal curing method (90 °C, 120 min), it can be seen that the average compressive strengths of the samples containing 10% CaCO_3_ were relatively close under the two curing methods. With 30% CaCO_3_, the average compressive strength of the microwave cured samples was slightly higher than that of the thermally cured sample. However, with 50% CaCO_3_, the average compressive strength of the microwave cured samples was much lower than that of the thermally cured sample (124 MPa), due to the presence of more pores in the structure. In order to further analyze the reasons for this phenomenon, the samples containing 50% CaCO_3_ were cured under 616 W microwave for 35 s. The average compressive strength of cured samples was about 74 MPa, which was higher than that of the samples cured under 800 W microwave for 30 s (18 MPa). Therefore, when the CaCO_3_ content was high, the compressive strength of the sample could be improved by appropriately reducing microwave power and prolonging microwave treatment time, thereby improving the compressive strength of the sample.

## 4. Conclusions

In this work, the microwave curing characteristics of UPR and its composites containing CaCO_3_ were studied. The main conclusions are listed below:

(1) The free-radical copolymerization reaction of the UPR sample occurred after 25 s of 800 W microwave treatment, faster than the thermal curing method at 90 °C (24 min). Under the two curing methods, the temperature of all the UPR samples need to exceed 65 °C before entering the stage of chain growth reactions. In addition, DSC and FTIR results showed that the curing degree and cured products of the UPR sample obtained via 30 s of microwave (800 W) were consistent with that obtained from 120 min of thermal curing (90 °C), indicating that microwave irradiation accelerated the curing rate of UPR without changing its curing effect.

(2) The addition of CaCO_3_ particles could make the samples enter the stages of chain growth reactions and chain termination more quickly during the curing process, which would shorten the curing time of the samples without changing the cured products of the UPR. However, increasing the addition of CaCO_3_ could lead to an increase in residual pore defectors in the UPR composites, thereby reducing their compressive strength. The defects could be improved by reducing the microwave power and prolonging the microwave treatment time. These results above would provide a new avenue for the microwave curing of the UPR composites.

## Figures and Tables

**Table 1:** Degree of cure for cured UPR samples.

Sample	*H* (J/g)	α (%)
Liquid UPR	234.50	
Microwave curing	13.62	94.19
Thermal curing	9.43	95.98

**Table 2:** Conversion rates of the C=C in UP under two curing methods.

Sample	1716 cm^−1^	981 cm^−1^	α_P_ (%)
Liquid UPR	2450	356	/
Microwave curing	2250	22	93.27
Thermal curing	2143	24	92.29

## Data Availability

The data presented in this study are available on request from the corresponding author.
